# Compliance with infection prevention and control standard precautions and factors associated with noncompliance among healthcare workers working in public hospitals in Addis Ababa, Ethiopia

**DOI:** 10.1186/s13756-024-01381-w

**Published:** 2024-03-13

**Authors:** Feyissa Regassa Senbato, Deneke Wolde, Merga Belina, Kehabtimer Shiferaw Kotiso, Girmay Medhin, Wondwossen Amogne, Tadesse Eguale

**Affiliations:** 1 Infection Prevention and Control Unit, Tikur Anbessa Specialized Hospital, College of Health Sciences, Addis Ababa University, P.O.Box 1176, Addis Ababa, Ethiopia; 2 Aklillu Lemma Institute of Pathobiology, Addis Ababa University, P.O.Box 1176, Addis Ababa, Ethiopia; 3 Department of Public Health, College of Medicine and Health Sciences, Worabe University, Worabe, Ethiopia; 4 Department of Medical Laboratory Science, College of Medicine and Health Sciences, Wachemo University, P.O.Box 667, Hosanna, Ethiopia; 5 Department of Statistics, College of Natural and Computational Sciences, Addis Ababa University, P.O.Box 1176, Addis Ababa, Ethiopia; 6 Department of Internal Medicine, College of Health Sciences, Addis Ababa University, P.O.Box 1176, Addis Ababa, Ethiopia; 7 The Ohio State University Global One Heath, Addis Ababa, Ethiopia; 8 Centre for Innovative Drug Development and Therapeutic Trials for Africa (CDT-Africa), College of Health Science, Addis Ababa University, Addis Ababa, Ethiopia; 9 Division of Epidemiology and Biostatistics, Department of Global Health, College of Medicine and Health Sciences, Cape Town, South Africa

**Keywords:** Compliance, Knowledge, Standard precautions, Infection Prevention, And Control

## Abstract

**Background:**

Standard Precautions (SPs) are the minimal infection prevention and control (IPC) measures that apply to all patient care activities at all times, regardless of whether the patient has a suspected or proven disease, in any place where healthcare service is provided. These evidence-based practices protect healthcare workers (HCWs) from infection while preventing the spread of infectious agents among patients, visitors, and the environment.

**Objectives:**

Assessed compliance of HCWs working in public hospitals in Addis Ababa to infection prevention and control SPs, and factors associated with noncompliance.

**Methods:**

In a hospital-based cross-sectional study, 422 HCWs were recruited from nine public hospitals in Addis Ababa using a stratified random sampling technique. Data were collected using self-administered questionnaires, entered into a computer using Epi data, and analyzed using SPSS version 25. The association between the independent and the outcome variables was investigated using logistic regression. Odd ratios with corresponding 95% confidence intervals (CI) were used as measures of the strength of the association between the outcome and the explanatory variables. A *p*-value below 5% was considered an indicator of statistical significance.

**Results:**

The level of knowledge of HCWs about IPC and SPs was 51.9% and 36.49% of the respondents were compliant with SPs. Receiving IPC Training [Adjusted Odds Ratio (AOR) = 1.81, 95% CI 1.06, 3.09], knowing SPs [AOR = 3.46, 95% CI = 1.83, 6.54], presence of a mechanism in the hospital to enforce the IPC practices [AOR = 1.71 95% CI = 1.01, 2.89], and availability of cleaning and disinfection chemicals in the hospital [AOR = 2.18, 95%CI = 1.15, 4.13] were significantly associated with the HCWs’ compliance with SPs.

**Conclusion:**

Compliance with IPC standard precautions of HCWs in public hospitals of Addis Ababa is suboptimal. Working in medical units, less work experience, lack of training, poor knowledge, absence of a mechanism to enforce adherence, and inadequate resources are independent predictors for non-compliance of the HCWs.

## Background

Healthcare-associated infections (HAIs) are major causes of preventable diseases, deaths, and increased healthcare costs. Many HAIs are caused by microorganisms that are present on the patient’s body (resident flora) or from transient sources, such as the hands of healthcare workers (HCWs’), contaminated equipment, or the environment [1]. Infection prevention and control (IPC) focuses on preventing avoidable infections in healthcare settings, safeguarding patients, healthcare workers, and visitors from harm. The spread of micro-organisms usually results from breaches in compliance with IPC standard precautions, such as inadequate hand hygiene and environmental cleaning, lapses in disinfection and sterilization, and incorrect use of personal protective equipment [2].

Over 59 million people work in healthcare facilities worldwide and they are regularly exposed to various health and safety risks [3]. The risks include exposure to various infectious agents [4]. A global report on infection prevention and control demonstrates that HAIs claim seven patients in high-income countries (HICs) and 15 patients in low- and middle-income countries (LMICs) and at least one HAI is acquired during a hospital stay for every 100 patients in acute-care hospitals [3].

The majority of HAIs can be avoided with easily accessible, reasonably priced IPC interventions like capacity building through training of HCWs on IPC basics, providing evidence-based guidelines, setting up management systems, providing IPC supplies and supportive supervision and mentoring [1].

Standard Precautions represent a set of evidence-based practices designed to protect healthcare personnel and patients from the transmission of infectious agents. At its core, Standard Precautions is an approach that assumes every individual, regardless of their apparent health status, could be carrying infectious agents. It treats all blood, body fluids, secretions, excretions (except sweat), non-intact skin, and mucous membranes as potentially infectious, necessitating a consistent application of preventive measures. These measures include hand hygiene, environmental cleaning and disinfection, personal protective equipment, healthcare waste management, safe injection practices, and respiratory hygiene, among others [5]. Since a considerable proportion of avoidable infections in healthcare settings are preventable through the basic application of standard precaution measures. It forms the foundation of infection control strategies, providing a baseline of protective measures that can be augmented based on the specific nature of patient care. The current study was designed to assess HCWs’ compliance with the standard precautions and identify factors associated with non-compliance.

## Methods

### Study setting and period

The study was conducted in Addis Ababa, the capital city of Ethiopia. Its average altitude is 2,400 m above sea level, with the highest elevation reaching 3,200 m, and one of the highest-altitude capital cities of the world [6].

Currently, Addis Ababa has 13 government and more than 40 privately owned hospitals. Out of the 13 hospitals, the Federal Ministry of Health administers four, two are administered under the army and police, five are administered under the city government of the Addis Ababa Health Bureau, and one (Tikur Anbessa Specialized Teaching Hospital) is administered by the Addis Ababa University.

Data for the current study were collected from October 2022 to January 2023, and the overall study period was from August 2022 to April 2023.

### Study design

An institutional-based cross-sectional study was employed.

### Sample size determination

The single population proportion formula, followed by proportional allocation [7], was used to calculate the required sample size with an assumption of a 95% confidence level, a margin of error of 5%, and a 10% non-response rate. This resulted in a final sample size of 422.

Nine government hospitals were selected using a simple random sampling method from the 13 Government-run hospitals in Addis Ababa (Tikur Anbessa Specialized Hospital, St Paul Millennium Medical College Hospital, Yekatit 12 Medical College Hospital, Zewditu Memorial Hospital, Minilik Referral Hospital, Gandhi Memorial Hospital, St Peter Referral Hospital, Alert Hospital, and Ras Desta Memorial Hospital). In each of these hospitals, healthcare workers were stratified into three categories: Physicians, Nurses, and Laboratory technologists. A list of the total number of healthcare workers (physicians, nurses, and laboratory technologists) was obtained from the Human Resource Departments of the respective hospitals.

Proportionate sample size allocation was used ${(n}_{f}={N}_{f}\times n/N)$ to determine the required number from individual professional categories in each of the study hospitals. Respondents were recruited into the study, using a random number table and then asked for informed consent to participate.

### Data collection methods and instruments

Structured, pre-coded, and self-administrated questionnaires were developed to collect sociodemographic characteristics, knowledge of standard precautions, compliance to standard precautions, and factors that could potentially be associated with the non-compliance of the healthcare workers. The data was collected using the paper-based form on a prepared questionnaire sheet. The validity and reliability of the developed questionnaires were tested. Before data collection, a pre-test was conducted on 10% of the total sample outside targeted hospitals. The value of Kuder-Richardson was 0.7, and the value of Cronbach’s alpha was 0.9, respectively. Data was collected following the necessary ethical guidance and protocols after receiving ethical approval from the Aklilu Lemma Institute of Pathobiology.

### Data analysis

Data were entered into a computer using EPI info version 5 after checking the completeness, accuracy, and clarity of collected data. SPSS version 25 software was used for statistical analysis of the data. Categorical variables were described using percentages, tables, or graphs whereas continuous variables were summarized using the mean (s.d) median (interquartile range). Ordinal logistic regression analysis was conducted to model the outcome variable. Independent variables with a *p*-value of less than 0.2 in bivariate analysis were entered into a multivariable logistic regression model to adjust for potential confounding effects of each of those variables while assessing the association of other variables. Multicollinearity among independent variables was checked using the variance inflation factor (VIF).

### Operational definitions

#### Knowledge

understanding of or having information about infection prevention and control that one can get through seminars, webinars, workshops, work experiences, and training. In the study, participants’ knowledge of Standard Precautions (SPs) was assessed through a set of questions covering different aspects of SPs. The participants were required to respond with either a “yes” or “no,” corresponding to numerical codes of 1 and 0, respectively. The responses to a total of 21 questions were then categorized into three distinct levels: optimal, suboptimal, and poor. This classification was based on the percentage scores achieved by each participant. Specifically, a participant’s knowledge was deemed optimal if their cumulative score exceeded 85%. Scores falling between 70% and 85% were classified as suboptimal, indicating a moderate level of knowledge. Conversely, scores below 70% were categorized as poor, suggesting a lower level of knowledge of the components of SPs [8].

#### Compliance

is a practical protocol that healthcare workers should follow during clinical care to prevent exposure to infection, antimicrobial resistance, and occupational infections from patients, healthcare workers, and the community. In the study, participant’s compliance with SPs was evaluated through a set of questions addressing different components of SPs. Response format of a 4-point adjectival scale, from never (0), seldom [1], sometimes [2], and always [3], was used. The total scores range from 0 to 22; a higher score indicates better compliance with standard precautions.

In the study, the assessment of a participant’s compliance with SPs involved a structured set of questions that delved into various components of SPs. The participants provided their responses using a 4-point adjectival scale, where each point was associated with a level of compliance: never (0), seldom [1], sometimes [2], and always [3]. The total scores on the compliance assessment ranged from 0 to 22. Compliance was considered optimal when scores exceeded 85%, between 70 and 85% were categorized as suboptimal, and scores below 70% were classified as poor [9].

#### Standard precautions

are the minimum infection prevention practices that apply to all patient care practices, regardless of the patient’s suspected or confirmed infection status, in any setting where healthcare services are delivered.

#### Infection prevention and control (IPC)

is a practical, evidence-based approach that prevents patients and healthcare workers from being harmed by avoidable infection and antimicrobial resistance.

#### Healthcare worker

a person with the proper education, training, and licensure to perform medical and surgical services. This term includes medical experts who use evidence-based practice care for patients, including physicians, nurses/midwifery laboratory technicians/technologists, health officers, and pharmacists/druggists.

## Results

### Sociodemographic characteristics of the study participants

The respondents’ median (IQR) age was 29.5 (27.0–34.0) years. Two hundred twenty-six (53.6%) of them were male and 228 (44%) were married. First-degree education was attended by 327 (77.5%) of the study participants, while 74 (17.5%) had attended master’s level education and above (Table 1).

**Table 1:** Socio-demographics characteristics of the study participants Addis Ababa, Ethiopia (*n* = 422)

Variables	Number	Percent
Age in year		
21–30	249	59.0
31–40	133	31.5
41–50	28	6.6
> 50	12	2.8
Sex		
Male	226	53.6
Female	196	46.4
Marital status of the respondent		
Single	188	44.5
Married	228	54.0
Other	6	1.4
The lowest level of education		
Diploma	21	5.0
First Degree	327	77.5
Masters and above	74	17.5

### Employment-related information among HCWs working in public hospitals

**Fig. 1:**
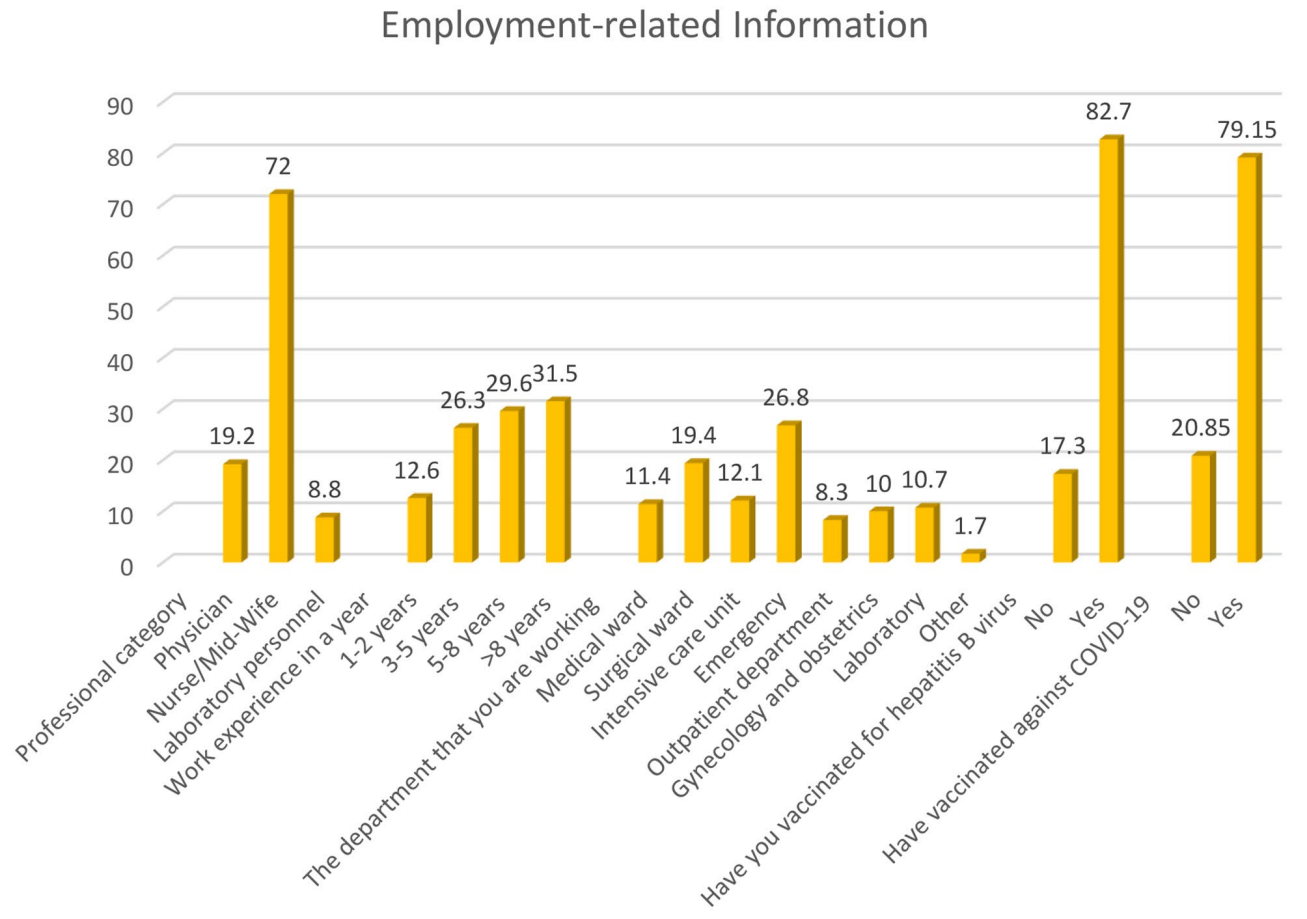
Employment-related information of the health care workers in public hospitals in Addis Ababa, Ethiopia 2023 (*n* = 422)

The majority of the study participants 304 (72%) were nurses/midwives, while physicians accounted for 81 (19.2%) of the total. Among healthcare workers, 113 (26.8%) worked in emergency units, and 82 (19.4%) worked in surgical wards. Furthermore, a significant portion of the participants, 349 (82.7%) were immunized against hepatitis B virus and 334 (79%) against COVID-19 (Fig. 1).

**Table 2:** Knowledge of infection prevention and control standard precautions of HCWs in public hospitals in Addis Ababa, Ethiopia 2023 (*n* = 422)

Measure of HCW’s Knowledge of Infection Prevention and Control Standard Precautions	HCWs Response	
	Yes n (%)	No n (%)
Do you consider every client and patient as potentially infectious or susceptible to infection?	379(89.8)	43 (10.2)
Standard precautions should be applied to all patients and clients attending the healthcare services	254(60.2)	168 (39.8)
Standard precaution is applied to all blood, body fluid, secretion, and execration except sweat, mucous membranes, and non-intact skin	306(72.5)	116 (27.5)
Hand hygiene is the single most important intervention for preventing the transmission of infections	376(89.1)	46 (10.9)
Do you know five moments of hand washing	348(82.5)	74 (17.5)
Hand washing is indicated between tasks and procedures on the same patient	318(75.4)	104 (24.6)
The use of alcohol-based antiseptic for hand hygiene is more effective than hand washing with plain soap and water	246(58.3)	176 (41.7)
The use of personal protective equipment relies on an HCW’s assessment of the risk of exposures/types, contact, and intensity of contact	343(81.3)	79 (18.7)
Use gloves before touching anything wet, such as non-intact skin, mucous membranes	384(91.0)	38 (9.0)
Hand washing should be practiced after the removal of the glove	334(79.1)	88 (20.9)
Personal protective equipment prevents the risk of acquiring infection	404(95.7)	18 (4.3)
The aseptic technique should be applied when preparing and delivering injections	373(88.4)	49 (11.6)
Used sharps and needles should be safely disposed of in closed and puncture-resistant containers	382(90.5)	40 (9.5%
Needles should be recapped after use	171(40.5)	251(59.5)
Patient care equipment should be cleaned between each use to prevent cross-contamination	380(90.0)	42(10.0)
Do you know the steps for reprocessing medical and surgical devices	326(77.3)	96 (22.7)
The frequently touched surface of the patient zone can harbor and cause the microorganism to spread across the facility via hands and contaminated items	371(87.9)	51 (12.1)
Do you know the different categories of waste and appropriate disposal methods at the point of waste generation?	315(74.6)	107(25.4)
Adherence to standard precautions prevents the occurrence of healthcare-associated infections	393(93.1)	29 (6.9)
Standard precaution contributes to preventing occupational infections among healthcare workers	372(88.2)	50 (11.8)
Compliance with infection prevention and control standard precautions plays an important role to prevent antimicrobial resistance	381(90.3)	41 (9.7)

### Healthcare workers’ knowledge of infection prevention and control standard precautions

The majority of study participants in the study, comprising 379 (89.8%) viewed every client and patient as potentially infectious or vulnerable to infection. Moreover, 254 (60%) believed that standard precautions should be applied for all individuals seeking healthcare services. Notably, only 306 (72.5%) of the study participants were aware that SPs should be applied for blood, body fluids, secretions, excretions (excluding sweat), mucous membranes, and non-intact skin.

About 376 (89%) of the study participants, knew that hand hygiene is the single most important intervention for preventing the spread of infections, and 348 (82.5%) were aware of the five moments of hand hygiene. Furthermore, 318 (75.4%) of the participants are aware that it is advisable to perform hand hygiene after handling the same patient for a while.

Regarding PPE, 343 (81.3%) are aware that an HCW must assess the circumstances before using it, and 384 (91.0%) are aware that an HCW should wear gloves if there is a chance that he/she will come in contact with non-intact skin, mucous membranes, or bodily fluids. To prevent needle-stick injuries, only 171 (40.5%) people are aware that needles should not be recapped after usage.

Most participants 380 (90.0%) knew that patient care equipment should be cleaned between each patient use to prevent cross-contamination. However, only 326 (77.3%)of participants knew the steps for reprocessing medical and surgical devices using sterilization or high-level disinfection, and 371 (87.9%) see the need to clean environmental surfaces around the patient in the patient care area to prevent infection.

Most participants 315 (74.6%) knew the importance of segregating infectious waste at the place where waste was generated, and 393 (93.1%) knew that adherence to standard precautions can prevent healthcare-associated infections. In addition, 381 (90.3%) of the study participants agreed that adherence to standard precautions could prevent the spread of antimicrobial resistance (Table 2).

**Table 3:** HCWs compliance with infection prevention and control standard precautions in public hospitals in Addis Ababa, Ethiopia 2023 (*n* = 422)

Level of compliance				
Items	Never N (%)	Seldom N (%)	Sometimes N (%)	Always N (%)
Wash/sanitize hands before touching a patient	49(11.6)	45(10.7)	189(44.8)	139(32.9)
Wash/sanitize hands before cleaning or aseptic procedures	28(6.6)	54(12.8)	148(35.1)	192(45.5)
Wash/sanitize hands after body fluid exposure	15(3.6)	13(3.1)	85(20.1)	309(73.2)
Wash/sanitize hands after touching a patient	23(5.5)	32(7.6)	171(40.5)	196(46.4)
Wash/sanitize hands immediately after removal of gloves	44(10.4)	49(11.6)	172(40.8)	157(37.2)
Wash/sanitize hands between patient contact	49(11.6)	66(15.6)	173(41.0)	134(31.8)
Wash/sanitize hands after touching the patient’s surroundings	31(7.3)	73(17.3)	173(41.0)	145(34.4)
When providing care considering that all patients as potentially infectious/susceptible to infection	25(5.9)	26(6.2)	164(38.9)	207(49.1)
I protect myself against the body fluids of all patients regardless of their diagnosis	10(2.4)	37(8.8)	116(27.5)	259(61.4)
I wear clean gloves whenever there is a possibility of exposure to anybody’s fluids	10(2.4)	23(5.5)	101(23.9)	288(68.2)
I change gloves between contacts with different patients	14(3.3)	32(7.6)	104(24.6)	272(64.5)
I avoid wearing my gown out of hospital compounds	41(9.7)	27(6.4)	64(15.2)	290(68.7)
I wear a waterproof apron whenever there is a possibility of body fluid splashing in my body	71(16.8)	56(13.3)	143(33.9)	152(36.0)
I wear eye goggles whenever there is a possibility of body fluid splashing in my face	94(22.3)	60(14.2)	124(29.4)	144(34.1)
I sterilize all reusable equipment before being used on another patient	27(6.4)	23(5.5)	98(23.2)	274(64.9)
I clean and disinfect equipment and environmental surfaces using detergents and disinfectants	36(8.5)	26(6.2)	116(27.5)	244(57.8)
I segregate non-infectious wastes in a black color-coded dust bin	22(5.2)	22(5.2)	124(29.4)	254(60.2)
I segregate infectious medical wastes in a yellow color-coded dust bin	23(5.5)	20(4.7)	133(26.8)	266(63.0)
I never bend needles with my hands	50(11.8)	16(3.8)	84(19.9)	272(64.5)
I avoid removing used needles from disposable syringes	40(9.5)	30(7.1)	108(25.6)	244(57.8)
I place used sharps in a puncture-resistant container at the point of use	30(7.1)	12(2.8)	93(22.0)	287(68.0)
I never recap needles	76(18.0)	43(10.2)	106(25.1)	197(46.7)

### Knowledge of various components of the standard precautions among different professional categories

Majority of physicians 63 (77.8%), nurses 227 (74.7%), and laboratory professionals 32 (86.5%) had a good level of knowledge of hand hygiene. Similarly, majority of professionals demonstrated a solid understanding of personal protective equipment (physician: 71 (87.7%), nurses: 265 (87.2%), laboratory professionals: 33 (87.2%). Furthermore, regarding safe injection practices (physicians: 73 (90.1%), nurses: 268 (87.2%), laboratory professionals: 33 (89.2%) have good knowledge.

Instrument reprocessing was the category with the lowest reported knowledge across all professional categories, with physicians reporting the lowest knowledge at 50 (61.7%). Cleaning and disinfection were well understood across all categories, with laboratory professionals 33 (89.2%) demonstrating the most knowledge, followed by nurses 269 (88.5%) and physicians 69 (85.2%).

**Fig. 2:**
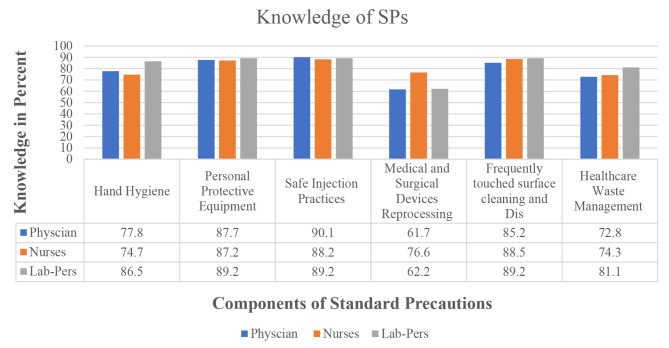
Knowledge of various components of the standard precaution among different professional categories in public hospitals Addis Ababa, Ethiopia 2023(*N* = 422)

Medical and surgical instrument reprocessing was generally well understood across all categories; 50 (72.8%) of physicians, 233 (74.3%) nurses, and 23 (81.1%) of laboratory professionals knew about reprocessing and similar proportions of all categories reported healthcare waste management (physicians: 59 (72.8%), nurses 226 (74.3%), laboratory professionals: 30 (81.1%) (Fig. 2).

### Healthcare workers’ compliance with infection prevention and control standard precautions

Table 3 demonstrates the HCWs’ level of compliance with different components of infection prevention and control standard precautions. One hundred eighty-nine (44.8%) and 139 (32.9%) of the HCWs reported that they wash/sanitize their hands before touching the patient “sometimes” and “always” respectively.

**Table 4:** Bivariate and multivariable ordinal logistic regression on factors associated with hcws compliance in public hospitals Addis Ababa, Ethiopia 2023 (*n* = 422)

Variables	Categories	COR (95% CI)	AOR (95% Cl)
Age	21–30	1.06(1.03, 1.09)*	1.05 (1, 1.1)**
Sex			
	Female	0.68(0.47, 0.96)*	0.8 (0.53, 1.22)
Marital status			
	Married	1.83(1.28, 2.63)*	1.21 (0.76, 1.95)
	Others	2.14(0.50, 8.99)	0.81 (0.12, 5.22)
Educational status			
	Degree	1.12(0.52, 2.39)	0.89 (0.36, 2.22)
	Master and above	3.47(1.47, 8.17)*	2.39 (0.85, 6.74)
Work Experience			
	3–5 years	1.99(1.06, 3.73)*	1.93 (0.92, 4.01)
	5–8 years	4.09(2.20, 7.58)*	2.43 (1.12, 5.27)**
	> 8 years	4.29(2.30, 7.97)*	0.95 (0.35, 2.56)
Department			
	Surgical ward	0.83(0.43, 1.61)	0.82 (0.38, 1.77)
	Intensive care unit	2.89(1.36, 6.13)*	2.63 (1.08, 6.44)**
	Emergency	1.02(0.55, 1.89)	0.97 (0.47, 2.03)
	OPD	2.00(0.89, 4.48)	2.57 (0.99, 6.66)
	Gyn&Obs	3.17(1.47, 6.81)*	3.87 (1.53, 9.75)**
	Laboratory	0.56(0.26, 1.22)	0.22 (0.03, 1.46)
	Others	0.92(0.20, 4.20)	0.61 (0.12, 3.13)
Profession			
	Nurses/Midwife	2.31(1.45, 3.68)*	2.3 (1.30, 4.04)**
	Laboratory personnel	0.97(0.46, 2.02)	4.21 (0.6, 29.48)
Training on IPC			
	Yes	1.85(1.24, 2.74)*	1.81 (1.06, 3.09)**
COVID-19 Vaccination			
	Yes	1.78(1.16, 2.74)*	1.62 (0.95, 2.75)
Knowledge of SPs			
	Sub-optimal	1.79(1.02, 3.12)*	1.58 (0.81, 3.07)
	Optimal	4.98(2.96, 8.37)*	3.46 (1.83, 6.54)**
Presence of guidelines on SPs			
	Yes	1.54(1.04, 2.27)*	0.67 (0.4, 1.13)
Presence of a mechanism to support/enforce IPC practice			
	Yes	2.66(1.78, 3.96)*	1.71 (1.01, 2.89)**
PPE availability			
	Yes, but not continuously available in sufficient quantities	1.47(0.96, 2.27)	1.32 (0.74, 2.35)
	Yes, continuously available in sufficient quantities	2.11(1.32, 3.37)*	1.34 (0.69, 2.6)
Presence of different bins per their types of waste			
	Separate bins present but more than ¾, only two bins (instead of three)	1.28(0.67, 2.47)	1.08 (0.49, 2.38)
	Yes	1.99(1.03, 3.83)*	1.95 (0.85, 4.45)
Water services availability all the time in sufficient quantities for all uses			
	Yes, available on average > 5 days/week/every day but not in sufficient quantity	1.07(0.72, 1.60)	0.62 (0.36, 1.08)
	Yes, every day and of sufficient quantity	1.78(1.12, 2.85)*	0.84 (0.43, 1.64)
Functioning hand hygiene stations (alcohol-based hand rub, soap, and water)			
	Yes, stations are present, but supplies are not reliably available	1.29(0.83, 2.02)	1.03 (0.56, 1.88)
	Yes, with reliably available resources	1.50(0.93, 2.43)	0.7 (0.34, 1.43)
Working environment flow pattern and activity controlled			
	Yes	1.53(1.07, 2.18)*	0.83 (0.51, 1.34)
Adequate and sustainable supply of cleaning and disinfecting agent			
	Yes, sometimes there is a disruption	2.08(1.28, 3.39)*	2.18 (1.15, 4.13)**
	Yes, always available	2.99(1.68, 5.33)*	1.75 (0.78, 3.95)
Adequate staffing			
	Yes	1.36(0.95, 1.95)	1.35 (0.84, 2.16)

Only 192 (45.5%) of the study participants “always” wash/sanitize their hands before performing clean or aseptic procedures. Most participants 309 (73.2%) “always” wash/sanitize their hands after exposure to body fluids.

Concerning was the fact that only 171(40.5%) of HCWs washed/sanitized hands ‘’sometimes’’ after touching the patients, and 196 (46.4%) indicated that they “always” sanitized/washed their hands when opportunities were there. About 172 (40.8%) of the HCWs washed/sanitized their hands “always” immediately after removing used gloves.

Regarding the washing/sanitizing of hands between patient contacts, 134 (31.8%) of the study participants are “always” compliant, and 173 (41.0%) washed/sanitized their hands “sometimes” after touching patients’ surroundings.

Two hundred seven (49%) of the HCWs “always” considered all patients potentially infectious/susceptible to infection during care. However, 259 (61.4%) of respondents reported, “always” protecting themselves using protective barriers regardless of the patient’s diagnosis. Two hundred and seventy-two (64.5%) of HCWs of all different professional categories indicated that they “always” changed gloves between different patients. Of these, 104 (24%) of them “sometimes” changed gloves between patients. Hundred fifty-two (36.0%) of the participants “always” wear plastic/rubber aprons whenever there is the possibility of body fluids splashing, and 143 (33.9%) of HCWs “sometimes” wear plastic/rubber aprons when indicated.

On reused, medical and surgical equipment cleaning and disinfection, and sterilization, 244 (57.8%) of respondents were “always” adhering to these practices. Two hundred seventy-four (64.8%) of the study participants were “always” compliant with cleaning and disinfecting frequently touched surfaces.

Compliance with healthcare waste management at the point of generation was relatively good with 254 (60.2%) and 266 (63.0%) respondents reporting compliance with waste segregation based on the contamination level and disposal into designated waste bins. Two hundred and seventy-two (64.5%) respondents indicated that they never bend needles by hand, avoiding contact with sharp boxes 244(57.8%), and 287(68.0%) of the participants were compliant with disposing of used needles and sharps into puncture-resistant boxes. However, only 197 (46.7%) of HCWs indicated that they never recap needles after each use (Table 3).

### Factors associated with Healthcare workers’ compliance with infection prevention and control standard precautions

In bivariable ordinal logistic regression sex, marital status, education status, work experience, department, profession, training in IPC, presence of mechanism to enforce/support IPC practices, PPE availability, presence of resources for waste disposal and segregation, water services, work environment flow pattern and activity, adequacy, and sustainability of cleaning and disinfection agents were identified as candidate variables for the multivariable analysis. However, after controlling the effects of confounding only, working experience, department, profession, IPC training, a mechanism to enforce IPC practices, and adequacy and sustainability of cleaning and disinfection were significantly associated with compliance with IPC standard precautions in multivariable ordinal logistic regression.

Healthcare workers who had accumulated 5–8 years of professional experience exhibited a higher likelihood of adhering to standard precautions, in comparison to HCWs with 3–5 years of experience. This increased adherence was significantly associated with optimal compliance, as indicated by the adjusted odds ratio (AOR = 2.43, 95% Cl = 1.12–5.27, *p* = 0.025. Likewise, HCWs in the gynecology and obstetrics department had four times the odds of compliance compared to those in the medical ward (AOR = 3.87, 95% CI = 1.53–9.75, *p* = 0.004). Nurses/midwives had higher odds than physicians of having optimal compliance (AOR = 2.29, 95% CI = 1.31–4.04, *p* = 0.004). HCWs who received IPC training were more likely to have sub-optimal and optimal compliance than those who did not (AOR = 1.813, 95% CI = 1.065–3.086, *p* = 0.028).

HCWs with optimal knowledge of standard precautions are more likely to comply with SPs than those with sub-optimal knowledge (AOR 3.46, 95% Cl = 1.83–6.54, *p* < 0.001). HCWs who work in hospitals with mechanisms to support/enforce IPC practice are more likely to comply with standard precautions than those who work in hospitals without such mechanisms (AOR = 1.71, 95% Cl = 1.01–2.89, *p* = 0.046).

Finally, when cleaning and disinfection agents were consistently provided, healthcare workers (HCWs) were more likely to comply with infection prevention and control standards (AOR = 2.18, 95% Cl = 1.15–4.13, *p* = 0.017) compared with when these chemicals were not (Table 4).

## Discussion

This cross-sectional study investigated the compliance of healthcare workers in public hospitals in Addis Ababa with SPs and factors associated with non-compliance to these principles. The level of compliance of HCWs with SPs was below the desired level, accounting only for 36.5%. The level of knowledge of HCWs regarding infection prevention and control SPs was favorable, with 51.9% having an optimal level of knowledge. Factors that positively influence the compliance level of HCWs were more years of work experience, being a nurse/midwife, having a better knowledge of SPs, the presence of mechanisms to enforce/support infection prevention and control practices, and the availability of sufficient and sustainable cleaning and disinfecting supplies.

In this study, the knowledge of infection prevention and control SPs among the HCWs was optimal. The result regarding knowledge of SPs is in line with earlier research carried out in Ethiopia [10–13]; nonetheless, reports of low-level knowledge of SPs have been reported in Ethiopia [14–16], Nigeria [17], and Saudi Arabia [18]. This discrepancy might be because of the methodological variations, the operational definition used in the recruited methodological analysis, and the facilities’ different natures.

The overall level of compliance with standard precautions in this study was sub-optimal (36.49%). The study finding is consistent with other research conducted in various regions of Ethiopia [11, 19, 20]. However, this finding shows higher compliance with SPs has been reported in Ethiopia [21, 22]. This study finding is lower than studies conducted in Ethiopia [23–25]. The discrepancy may be due to methodological variations and scoring differences.

In this study, HCWs having working experience of ≥ 5 years had 2.5 times higher odds of complying with infection prevention and control SPs than those with less than five years of working experience. This finding was consistent with other studies from Bahir Dar and Debre Tabor [20, 26] but other studies from Brazil indicated that HCWs with more than 20 years of work experience complied more with SPs than those below 20 years of working experience [27].

Experience at a senior level can be pivotal in ensuring that HCWs comprehend the potential risks and hazards associated with their duties. It also contributes to their understanding of the necessary safety protocols and measures that need to be implemented to mitigate these risks. In addition, a more plausible explanation would be that senior HCWs had a chance to participate in different knowledge-empowering seminars, workshops, conferences, and training. HCWs working in the intensive care unit, gynecology, and obstetrics wards are three to four times more compliant with SPs than those working in a medical ward.

The study from Tanzania was consistent with this study, implying HCWs working in intensive care units had more practice with SPs when compared with those participants working in emergency and recovery rooms [28].

Even though the degree of association is not mentioned, another study from the Woliyata zone, Ethiopia stated that working place/department and level of compliance were significantly associated with compliance of HCWs to SP measures [29]. This could be due to HCWs working in the ICU and obstetrics and gynecology (OBGYN) departments being more likely to encounter patients with infectious diseases and conditions. They may be more aware of the potential risks and consequences of not following SPs and may, therefore, be more diligent in adhering to them.

The finding of this study revealed that nurses/midwives had 2.3 times the odds of adhering more to infection prevention and control SPs measures compared with physicians. This study is in agreement with other studies from Italy, Brazil, Ethiopia, and Nigeria [11, 24, 30–32].

Contrary to this finding studies from Northern Ethiopia show laboratory professionals are more compliant with SPs than nurses/midwives and physicians [19].

The possible explanation for the finding is that laboratory professionals exhibit higher compliance with SPs because they often receive specialized training that emphasizes strict adherence to safety protocols due to the handling of potentially hazardous materials, such as biological specimens, sharps, and chemicals.

HCWs who received Infection prevention and control training were almost two times more compliant with infection prevention and control SPs than those not receiving training. Many studies support these findings and demonstrate that HCWs who participated in infection prevention and control training were more compliant with SPs [16, 24, 25, 27, 28]. One study in Ethiopia was however not in agreement with this study [33].

The differing findings between studies could be due to variations in the training programs themselves (e.g., contact, duration delivery methods), healthcare settings or population studied, and variations in the measurement of compliance.

Based on the study, it was found that HCWs who had an optimal level of knowledge of SPs had 3.5 times the odds of compliance with to adhere with SPs compared with HCWs who had a suboptimal level of knowledge. Other studies are in line with the findings [10, 34]. However, other studies do not reveal findings similar to this study and could not find any proof that HCWs who are knowledgeable about SPs comply more than those with poor knowledge [30, 32]. The discrepancy in findings regarding the association between knowledgeable healthcare workers (HCWs). Variations in the tools, questionnaires, or criteria used to evaluate knowledge and compliance could contribute to differences in the findings.

The presence of a mechanism to enforce/support IPC practices made HCWs nearly twice as adherent with SPs compared to when there is no support for IPC practices. The previous studies conducted in Addis Ababa and Hawassa were congruent with this study [23, 24].

Immediate feedback on infection prevention and control (IPC) practices to healthcare workers (HCWs) according to WHO and CDC recommendations can have a significant positive impact. HCWs who receive immediate feedback are more likely to improve their IPC practices and adhere to standard precautions, leading to reduced transmission of infections.

Immediate feedback can identify gaps in knowledge or areas where IPC practices need improvement, allowing targeted education and training to address these issues. By implementing immediate feedback mechanisms, healthcare facilities can promote a culture of continuous improvement in IPC practices and ultimately improve patient safety [3].

Regarding the adequacy and sustainability of cleaning and disinfection supply, HCWs reported that having those chemicals is associated with 2.18 times the odds of compliance with SPs than those without adequate supplies. According to the WHO 2022 global report on IPC, adequate and sustainable resources for cleaning and disinfection purposes support the HCW’s compliance with SPs. Studies from Debremarkos and Rwanda support the findings of this study [35, 36]. One of the study limitations was the possibility of response bias as study participants likely over-report their practices.

## Conclusion

The findings of this study demonstrated that the overall compliance with SPs was suboptimal and HCWs’ knowledge of infection prevention and control SPs was optimal. This suggests that there is room for improvement in the compliance of healthcare workers with infection prevention and control standard precautions (SPs) in public hospitals in Addis Ababa, Ethiopia. The study identified factors such as having extensive working experience, working in the ICU and OBGYN departments, receiving infection prevention and control training, having good knowledge of SPs, and having a mechanism to enforce/support IPC practices that are positively associated with compliance with SPs. Hence, continuous education, training, and reinforcement should be conducted to sustain compliance and address gaps observed in this study. There is also a need for the development of clear guidelines and standard operating procedures for infection prevention and control within healthcare facilities and the adequate availability of resources and supplies required for the implementation of IPC activities.

## Data Availability

All data generated or analyzed during this study are included in this published article [and its supplementary information files] Original data are available from the corresponding author upon reasonable request.

## Abbreviations

IQR: Interquartile Range
HCW: Healthcare Worker
IPC: Infection Prevention and Control
PPE: Personal Protective Equipment
SPs: Standard Precautions
AOR: Adjusted Odds Ratio
COR: Crude Odds Ratio
CI: Confidence Interval
OPD: Outpatient Department
CDC: Communicable Disease Center
ICU: Intensive Care Unit
OBGYN: Obstetrics and Gynecology
WHO: World Health Organization
